# Biochemical profiles and organ dysfunction in neonates with hypoxic-ischemic encephalopathy post-hoc analysis of the THIN trial

**DOI:** 10.1186/s12887-024-04523-6

**Published:** 2024-01-15

**Authors:** Karen Haugvik Francke, Ragnhild Støen, Niranjan Thomas, Karoline Aker

**Affiliations:** 1 Faculty of Medicine and Health Science, Norwegian University of Science and Technology (NTNU), Trondheim, Norway; 2 Department of Pediatrics, St. Olavs Hospital, Trondheim University Hospital, Trondheim, Norway; 3 Department of Clinical and Molecular Medicine, NTNU, Trondheim, Norway; 4 Department of Neonatology, Christian Medical College, Vellore, India; 5 Department of Neonatology, Joan Kirner Women’s and Children’s at Sunshine Hospital, Melbourne, 3021 Australia; 6 Norwegian University of Science and Technology (NTNU), Trondheim, Norway

**Keywords:** Therapeutic hypothermia, Biochemical profiles, Hypoxic-ischemic encephalopathy

## Abstract

**Background:**

Therapeutic hypothermia for infants with moderate to severe hypoxic-ischemic encephalopathy is well established as standard of care in high-income countries. Trials from low- and middle-income countries have shown contradictory results, and variations in the level of intensive care provided may partly explain these differences. We wished to evaluate biochemical profiles and clinical markers of organ dysfunction in cooled and non-cooled infants with moderate/severe hypoxic-ischemic encephalopathy.

**Methods:**

This secondary analysis of the THIN (Therapeutic Hypothermia in India) study, a single center randomized controlled trial, included 50 infants with moderate to severe hypoxic-ischemic encephalopathy randomized to therapeutic hypothermia (*n* = 25) or standard care with normothermia (*n* = 25) between September 2013 and October 2015. Data were collected prospectively and compared by randomization groups. Main outcomes were metabolic acidosis, coagulopathies, renal function, and supportive treatments during the intervention.

**Results:**

Cooled infants had lower pH than non-cooled infants at 6–12 h (median (IQR) 7.28 (7.20–7.32) vs 7.36 (7.31–7.40), respectively, *p* = 0.003) and 12–24 h (median (IQR) 7.30 (7.24–7.35) vs 7.41 (7.37–7.43), respectively, *p* < 0.001). Thrombocytopenia (< 100 000) was, though not statistically significant, twice as common in cooled compared to non-cooled infants (4/25 (16%) and 2/25 (8%), respectively, *p* = 0.67).

No significant difference was found in the use of vasopressors (14/25 (56%) and 17/25 (68%), *p* = 0.38), intravenous bicarbonate (5/25 (20%) and 3/25 (12%), *p* = 0.70) or treatment with fresh frozen plasma (10/25 (40%) and 8/25 (32%), *p* = 0.56)) in cooled and non-cooled infants, respectively. Urine output < 1 ml/kg/h was less common in cooled infants compared to non-cooled infants at 0–24 h (7/25 (28%) vs. 16/23 (70%) respectively, *p* = 0.004).

**Conclusions:**

This post hoc analysis of the THIN study support that cooling of infants with hypoxic-ischemic encephalopathy in a level III neonatal intensive care unit in India was safe. Cooled infants had slightly lower pH, but better renal function during the first day compared to non-cooled infants. More research is needed to identify the necessary level of intensive care during cooling to guide further implementation of this neuroprotective treatment in low-resource settings.

**Trial registration:**

Data from this article was collected during the THIN-study (Therapeutic Hypothermia in India; ref. CTRI/2013/05/003693 Clinical Trials Registry – India).

**Supplementary Information:**

The online version contains supplementary material available at 10.1186/s12887-024-04523-6.

## Introduction

Therapeutic hypothermia (TH) for infants with moderate to severe hypoxic-ischemic encephalopathy (HIE) reduces mortality (relative risk 0.75 (95% CI, 0.64–0.88)) and long-term disability (relative risk 0.77 (95% CI, 0.63–0.94)) [1], and has been standard of care in high income countries (HICs) since 2010 [2]. Although controversy remains whether TH should be provided in low- and middle-income countries (LMICs) [3–6], the latest international guidelines on Neonatal Life Support recommends implementation of TH in such settings if certain intensive care facilities like intravenous therapy, respiratory support, pulse oximetry, antibiotics, and anticonvulsants are available [7]. A recent systematic review and meta-analysis including 2926 infants reported reduction in disability and cerebral palsy (CP) in infancy by TH independent of setting, but a reduction in mortality at 18–24 months was only reported in HICs [8].

The largest randomized controlled trial (RCT) on TH till date, the Hypothermia for Encephalopathy in Low- and Middle-Income Countries (HELIX) trial, including 408 infants from India, Bangladesh and Sri Lanka [9], found increased mortality among cooled infants. Cooled infants had more complications such as persistent hypotension and metabolic acidosis, prolonged blood coagulation, gastric bleeding, and severe thrombocytopenia, suggesting that cooling in those settings had adverse effects potentially contributing to the increased mortality. Such effects of a moderate decrease in core temperature are consistent with what has been found in studies of accidental hypothermia [10–12]. Moderate hypothermia may affect cardiac, liver and renal function, but such organ complications are difficult to distinguish from the effects of a hypoxic-ischemic insult itself [13, 14]. Studies on TH for HIE from HICs has not reported any difference in the occurrence of hypotension, hemorrhage, or coagulopathies [15–19].

There are few studies with a normothermic control group after the implementation of TH as standard of care for infants with moderate to severe HIE. Detailed biochemical and clinical markers of organ dysfunction during TH are important to understand the conflicting results on adverse outcomes, safety, and efficacy from different settings and to optimize the treatment across settings. Although guidelines recommend that TH is provided in facilities with a certain level of intensive care [7, 20, 21], the level of monitoring and supportive treatment necessary for TH to be both effective and safe is still unclear.

The Therapeutic Hypothermia in India (THIN) study was a prospective RCT in which infants with moderate or severe HIE were randomized to TH or standard care with normothermia [22]. Both the primary outcome of early brain MRI biomarkers and secondary outcome of neurodevelopment at 18 months showed a beneficial effect of TH [22, 23]. The main purpose of this post-hoc analysis is to compare early biochemical profile and organ complications in cooled and non-cooled infants included in the THIN study. We also explore associations between organ dysfunction in the neonatal period and outcomes at 18 months.

## Methods

This is secondary analysis of the THIN study, a single-center RCT of infants admitted with moderate to severe HIE to the neonatal intensive care unit (NICU) at the Christian Medical College Vellore, a tertiary care teaching hospital in rural south India. Infants at or near term (> 35 weeks of gestation) admitted before 5 h after birth with signs of perinatal asphyxia (5-min Apgar-score < 6, pH < 7.0 base deficit ≥12, need of positive pressure ventilation > 10 min, or for outborn infants; no cry at birth) and moderate to severe HIE (identical to the NICHD trial [17]) were recruited between September 2013 and October 2015. Included infants were randomly assigned to hypothermia with target core temperature 33.5 °C ± 0.5 °C for 72 h induced by a phase changing material-based cooling device (MiraCradle Neonate Cooler, Pluss Advanced Technologies, India) or standard care (SC) with normothermia. A sample size of 25 infants in each arm of the RCT was calculated to detect a 10% difference in mean fractional anisotropy (FA) values in posterior limb of the internal capsule (PLIC) on neonatal MRI, and accounting for a 20% mortality before MRI was taken. Full description of the trial is published elsewhere [22].

For this study, we included biochemical data, clinical indicators of organ dysfunction, treatment data, seizures, adverse events during the intervention and neurodevelopmental outcome at 18 months. Blood gas, renal and coagulation parameters and full blood counts were monitored per study protocol, and investigation for infection was done as clinically indicated. Blood gas from cord or the infant within the first hour of life were only available in inborn infants. Persistent metabolic acidosis was defined as pH < 7.15 for more than 12 h. Liver enzyme analysis was only performed once, prior to starting TH and were thus not included in the analysis. Abnormal international normalized ratio (INR) and activated partial thromboplastin time (APTT) were defined as > 1.8 and > 43 s, respectively [24]. Thrombocytopenia was defined as platelet count < 100 000 per µl and severe thrombocytopenia as platelet count < 25 000 per µl or < 50 000 per µl with active bleeding. Anuria was defined as urine output < 0.5 ml/kg/h, and oliguria as urine output < 1 ml/kg/h.

All treatments including medications were given as per existing treatment protocols. Mechanical ventilation was provided for infants with respiratory failure. First-line anticonvulsants was phenobarbitone, second was phenytoin, and third and fourth were levetiracetam and benzodiazepine (midazolam or clonazepam). Infants were followed up at 18 months with a complete neurological examination and the Bayley Scales of Infant and Toddler Development, third edition (Bayley-III) [25]. Adverse outcome was defined as death, CP with GMFCS (Gross Motor Function Classification System) level 3–5 or Bayley-III cognitive and/or motor composite score (CS) < 85 at 18 months of age [23].

### Statistical analysis

All statistical analysis were performed using IBM SPSS Statistics 27 and 29. The data is presented as counts with proportions for dichotomous variables and median with IQR for continuous variables, as we had a small sample size, and did not expect the variables to be normally distributed. Group differences by randomization and outcome were analyzed using Chi^2^-test, Fisher’s exact test and linear-by-linear association as appropriate for dichotomous variables and Mann Whitney U-test for continuous variables. A *p*-value < 0.05 was considered statistically significant for all analysis. Unadjusted odds ratios for an adverse outcome were calculated for significant exposures.

## Results

Fifty infants were included in the THIN study, 25 receiving TH and 25 receiving SC. Demographics, neonatal characteristics and outcome are shown in eTable 1 in Supplement.

**Fig. 1:**
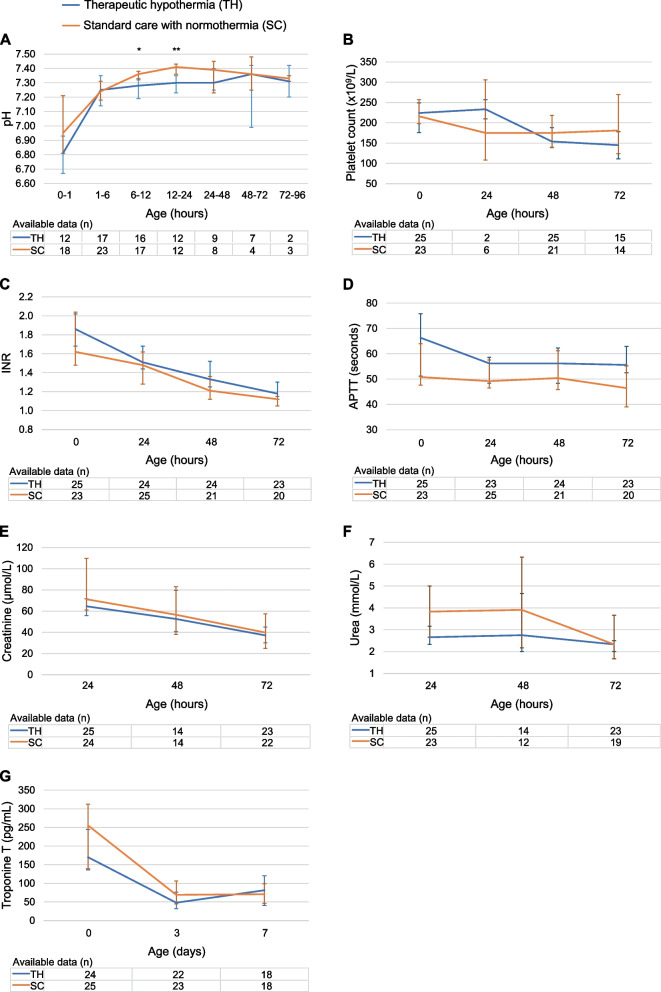
Trendlines for biochemical data in cooled and non-cooled infants. Figure shows median (95% CI) pH (**A**), platelet count (**B**), international normalized ratio (INR) (**C**), activated partial thromboplastin time (APTT) (**D**), creatinine (**E**), urea (**F**) and troponin T (**G**). Number of infants with available data is given in the x-axis. *p = 0.003, **p < 0.001. Abbreviations. TH, therapeutic hypothermia; SC, standard care with normothermia

Longitudinal biochemical data on pH, platelet count, INR, APTT, creatinine, urea and troponin T in the TH- and SC-group are shown in Fig. 1. Infants in the TH-group had significantly lower pH at 6–12 h compared to the SC-group (median (IQR) 7.28 (7.20–7.32) vs 7.36 (7.31–7.40), respectively, *p* = 0.003) and 12–24 h (median (IQR) 7.30 (7.24–7.35) vs 7.41 (7.37–7.43), respectively, *p* < 0.001). No infant had a persisting pH-value < 7.15 for more than 12 h.

Data on biochemical profile, organ dysfunction and treatment according to randomization group are presented in Table 1. During the first 24 h of life, significantly more infants in the SC-group had anuria/oliguria, compared to the TH-group (16/23 (70%) vs 7/25 (28%), respectively; *p* = 0.004, Table 1). This difference persisted also when excluding the four infants (all in SC-group) with global brain injury on MRI. Fourteen (28%) infants received mechanical ventilation with a maximum duration of three days, and two of these (one in TH and one in SC group) were on high frequency oscillation. All, except one infant in SC-group, were started on empirical antibiotic therapy, and twenty-one (11 in TH- and 10 in SC-group) were treated for more than 72 h. No infant had culture-positive sepsis.

**Table 1:** Biochemical data, organ dysfunction and treatment during admission

Randomization	Therapeutic hypothermia (*n* = 25)	Standard care (*n* = 25)	Total (*n* = 50)	*p*-value
**Biochemical data**				
Max Creatinine (µmol/L), median (IQR), [range]	65 (54–82) [39–194]	73 (55–111) [31–314]	68 (55–87) [31–314]	0.35
Max Urea (mmol/L), median (IQR), [range]	3.0 (2.5–4.3) [1.8–7.3]	3.8 (2.5–6.5) [1.5–11.3]	3.2 (2.5–5.0) [1.5–11.3]	0.17
Max CRP (mg/dL), median (IQR) [range]^a^	8.6 (4.2–26.1) [0.0–80.1]	10.0 (5.3–17.9) [0.0–66.9]	8.6 (4.3–21.3) [0.0–80.1]	0.92
Max Troponin T (pg/mL), median (IQR), [range]	170 (109–257) [59–841]	256 (126–345) [75–1180]	193 (126–310) [59–1180]	0.24
Platelets				
Minimum platelet count (× 10^9^/L), median (IQR), [range]	153 (127–177) [54–252]	169 (110–219) [39–325]	160.5 (121–189) [39–325]	0.19
< 100 000 per µl, n/N(%)^b^	4/25(16)	2/25(8)	6/50(12)	0.67
INR				
Max INR, median (IQR), [range]	1.9 (1.6–2.4) [1.4–4.9]	1.7 (1.5–2.5) [1.1–5.2]	1.8 (1.5–2.4) [1.1–5.2]	0.28
> 1.8, n/N (%)	15/25 (60)	10/25 (40)	25/50 (50)	0.16
APTT				
Max APTT (sec), median (IQR), [range]	74 (63–85) [44–180]	61 (49–84) [34–179]	68 (58–85) [34–180]	0.097
> 43 s, n/N (%)	25/25 (100)	23/25 (92)	48/50 (96)	0.49
**Organ dysfunction and treatment**				
Anuria/oliguria, n/N (%)^c^				
0–24 hours^d^	7/25 (28)	16/23 (70)	23/48 (52)	0.004
24–48 h	1/23 (4)	5/23 (22)	6/46 (13)	0.19
48–72 h	0/22 (0)	1/19 (5)	1/41 (2)	0.46
> 72 h	0/21 (0)	1/20 (5)	1/41 (2)	0.49
Number of vasopressors				0.38^e^
0	11/25 (44)	8/25 (32)	19/50 (38)	
1	10/25 (40)	12/25 (48)	22/50 (44)	
2 or more	4/25 (16)	5/25 (20)	9/50 (18)	
Intravenous bicarbonate, n/N(%)	5/25(20)	3/25 (12)	8/50(16)	0.70
Highest level of respiratory support, n/N (%)				0.53^f^
Intermittent mandatory ventilation	5/25 (20)	7/25 (28)	12/50 (24)	
High frequency oscillation	1/25 (4)	1/25 (4)	2/50 (4)	
Duration of antibiotics (days), median (IQR) [range]^g^	3 (3–5.75) [2–15]	3 (3–5) [2–7]	3 (3–5) [2–15]	0.501
Clinical seizures, n/N (%)	20/25 (80)	22/25 (88)	42/50 (84)	0.70
Number of anticonvulsants, n/N (%)				0.17
0	6/25 (24)	3/25 (12)	9/50 (18)	
1	10/25 (40)	10/25 (40)	20/50 (40)	
2	6/25 (24)	6/25 (24)	12/50 (24)	
3	3/25 (12)	5/25 (20)	8/50 (16)	
4	0/25 (0)	1/25 (4)	1/50 (2)	
Any blood products, n/N (%)	10/25 (40)	9/25(36)	19/50 (38)	0.77

*Abbreviations*. *INR* International normalized ratio, *APTT* Activated partial thromboplastin time

^a^Data was unavailable for 1 infant in the TH-group and 1 in the SC-group. ^b^Only 1 infant had a platelet count < 50 000 per µl; this infant had a platelet count of 39 000 and platelet clumps seen in smear, but no active bleeding. ^c^Anuria was defined as urine output < 0,5 ml/kg/h. Oliguria was defined as urine output < 1 ml/kg/h. ^d^A minimum of 12 h within the first 24 h of life. ^e^P-value based on Chi2-test on infants who received treatment with vasopressors vs. infants who did not. ^f^P-value based on Chi^2^-test on infants who were treated with mechanical ventilation vs. infants not treated with mechanical ventilation. ^g^Data was unavailable for 1 infant in the TH-group and 1 in the SC-group. 1 infant in the SC-group was not treated with antibiotics and is not included in estimation of median and IQR

No infant had severe thrombocytopenia. Fresh frozen plasma was the most frequently used blood product during the intervention (10/25 (40%) in the TH-group and 8/25 (32%) in the SC-group, *p* = 0.56). Only two infants in the TH-group and one in the SC-group received packed red blood cells, and none were given a platelet transfusion. Two infants (SC-group) had subgaleal bleeds. No infants in the study had subcutaneous fat necrosis.

Anuria/oliguria at 24–48 h, treatment with vasopressors, mechanical ventilation and ≥ 2 anticonvulsants were all significantly associated with an adverse outcome (Table 2).

**Table 2:** Organ dysfunction and treatments by outcome at 18 months^a^

Outcome	Good (*n* = 30)	Adverse (*n* = 17)	*p*-value	OR	CI (95%)
Anuria/oliguria 24–48 h, n/N (%)^b^	1/27 (4)	5/16 (31)	0.02	11.82	1.23, 113.23
Vasopressors, n/N (%)	14/30 (46)	14/17 (82)	0.017	5.33	1.27, 22.48
Mechanical ventilation, n/N (%)	3/30 (10)	8/17 (47)	0.009	8.00	1.74, 36.81
≥ 2 anticonvulsants, n/N (%)	8/30 (27)	13/17 (77)	< 0.001	8.94	2.24, 35.61

^a^Adverse outcome was defined as Bayley Scales of Infant and Toddler Development, third edition, cognitive and/or motor composite score < 85 (motor/cogn comp score), cerebral palsy with Gross Motor Function Classification System level 3–5, or death. 3 infants were lost to follow-up. ^b^Anuria/oliguria was defined as urine output < 1 ml/kg/h

## Discussion

This is a secondary analysis of the THIN study, an RCT of cooling induced by phase changing material versus standard care for infants with moderate or severe HIE admitted to a level III NICU in South-India. We report that cooled infants had lower pH during the first day of life compared to non-cooled infants, but no infant had persistent metabolic acidosis. Fewer cooled infants had early oliguria/anuria. There were no differences in other organ complications or in respiratory and hemodynamic support between cooled and non-cooled infants. These findings are consistent with those from HICs, showing low rates of adverse events during cooling [15–19].

Our finding that no infant had persistent metabolic acidosis is in in contrast to the HELIX-study, where 23% of cooled and 12% of non-cooled infants had persistent metabolic acidosis [9]. Hemodynamic control with close monitoring of blood pressure is key to avoid persistent hypotension and metabolic acidosis due to poor perfusion during TH [10, 26]. All infants in the THIN study had arterial line for continuous monitoring, and cardiac ultrasound to assess hemodynamics was available as standard of care. Blood pressure data were unfortunately not collected, but there was no difference in the use of vasopressors between the groups. The HELIX-trial reports significantly more use of pressors and more persistent hypotension despite maximum inotropic support among cooled infants [9, 27]. Similar to our findings, large RCTs from HICs have not reported an increased risk of hypotension or persistent metabolic acidosis with TH [15–19], and this could reflect the quality of intensive care and monitoring provided [26].

A higher, although not statistically significant, incidence of thrombocytopenia among cooled compared to non-cooled infants in the present study, is in line with what has been reported by others [5, 14, 28, 29]. Similarly, more cooled infants had elevated INR, but this difference was also not significant. Both groups had a very high proportion of infants with elevated APTT. This is most likely due to samples taken from arterial lines running heparin, which could interfere with the actual levels even if protocol was to withdraw at least 5 mL of blood before taking samples. More importantly, cooled infants did not have increased incidence of severe bleeding or other clinical indicators of severe coagulopathies, which is in line with studies from HICs [15–19]. This is also in contrast to the HELIX-study, where severe thrombocytopenia, prolonged blood coagulation and gastric bleeds were more frequent in the TH-group [9]. A high proportion of infants in both groups received fresh frozen plasma, reflecting the use of plasma to correct biochemical abnormalities in coagulation status.

Renal dysfunction is a common complication after perinatal asphyxia [30]. We found less anuria/oliguria in cooled than non-cooled infants, even after excluding four infants in the SC-group with global brain injury on MRI. Even though four large RCTs on cooling in HICs did not report any significant differences in renal dysfunction [15–18], the possible reno-protective effect of TH in our study is supported by a meta-analysis reporting significantly less acute kidney injury in cooled compared to non-cooled infants [31]. A recent cohort-study with a low incidence of acute kidney injury among cooled neonates with HIE also supports a reno-protective effect of TH [32].

No infant in our study had culture-proven sepsis, and there was no significant difference between the groups in infection parameters. Unit protocol was to start antibiotics in all cases without an obvious sentinel event as cause of HIE. Antibiotics was also continued beyond 72 h if CRP was still elevated. Cooling may lead to a late peak in CRP in the absence of infection [33], and the use and duration of prophylactic antibiotics in infants with HIE undergoing TH is still a matter of controversy [34]. A high rate of perinatal infection in LMICs has been suggested as a possible reason that TH may not be effective [35–37]. This is not supported by our data, and recent studies have reported similar mortality and a beneficial effect on neurodevelopment with TH, even in the presence of infection [38].

The THIN study has reported improved outcome with TH and these new findings of similar biochemical profiles and level of supportive treatments, support that this treatment is both effective and safe in our setting. A recent Indian cohort study on 155 cooled infants reports a higher incidence of sepsis and more use of invasive ventilation than in our trial, but argues that most cases are manageable in well-equipped NICUs [32]. Although there is no clear consensus on the optimal supportive care for infants receiving TH in any setting, respiratory support, continuous monitoring and preservation of hemodynamic stability, 24/7 imaging services etc. are standard in cooling centers in HICs [7, 21]. These requirements pose a challenge in many low-resource settings, where the access to neonatal intensive care and transport services vary greatly. Clinical guidelines and recommendations for supportive treatments, as well as organization of services including transport, are needed and should be the focus of future research in both HICs and LMICs.

The main limitation of this study is the small sample size. This limits the possibility to make prediction models for outcome based on organ dysfunctions and/or biochemical profiles [39]. We did not have data on blood pressure and some of the biochemical measurements were missing for many infants, especially pH-values. The generalizability of the findings to other populations and settings is unclear. Finally, the data in this study are a post-hoc analysis of an RCT, which is a limitation and require caution when interpreting the results. Despite these limitations, we believe our study supports that the safety and efficacy of TH depends on the level of intensive care available and not population differences as previously suggested [9, 40, 41].

## Conclusions

The findings in this post hoc analysis of a single-center RCT from India suggest that cooling for infants with moderate to severe HIE was not associated with more neonatal morbidities compared to standard care with normothermia. Despite lower pH at two time intervals, our findings do not support a negative effect of cooling on organ function in this setting. This suggests that the level of intensive care provided during TH may explain the lack of safety which have been reported in some studies from LMICs.

### Supplementary Information

**Additional file 1: eTable 1.** Demographics, neonatal characteristics and outcome. 

## Data Availability

The datasets used and/or analyzed during the current study are available from the corresponding author on reasonable request. Anonymized data (including data dictionaries) will be made available on request to researchers who provide a methodologically sound proposal for use in achieving the goals of the approved proposal.

## Abbreviations

APTT: Activated partial thromboplastin time
CP: Cerebral palsy
HELIX: Hypothermia for encephalopathy in low- and middle-income countries
HICs: High-income countries
HIE: Hypoxic-ischemic encephalopathy
INR: International normalized ratio
LMICs: Low- and middle-income countries
NICU: Neonatal intensive care unit
RCT: Randomized controlled trial
SC: Standard care with normothermia
TH: Therapeutic hypothermia
THIN: Therapeutic hypothermia in India
