# Supplementary material for: Biochemical profiles and organ dysfunction in neonates with hypoxic-ischemic encephalopathy post-hoc analysis of the THIN trial
Source: BMC Pediatr. 2024 Jan 15;24:46. doi: 10.1186/s12887-024-04523-6 (PMC10789058; doi:10.1186/s12887-024-04523-6)
Supplement: Supplementary file 1 — Additional file 1: eTable 1. Demographics, neonatal characteristics and outcome. [file 12887_2024_4523_MOESM1_ESM.docx]

**Supplement**

**eTable 1: Demographics, neonatal characteristics and outcome**

|  | Therapeutic hypothermia (n=25) | Standard care (n=25) | Total (n=50) |
| --- | --- | --- | --- |
| Inborn, n/N (%) | 13/25(52) | 17/25(68) | 30/50 (60) |
| Mode of delivery, n/N (%) |  |  |  |
| Normal vaginal | 9/25(36) | 10/25(40)^a^ | 19/50 (38) |
| Operative vaginal | 5/25 (20) | 5/25 (20) | 10/50 (20) |
| Cesarean section | 11/25 (44) | 10/25 (40) | 21/50 (42) |
| Male gender, n/N (%) | 17/25 (68) | 16/25 (64) | 33/50 (66) |
| GA (weeks), mean (SD) | 39.1(1.3) | 39.2 (1.4)^b^ | 39.1(1.3) |
| Birth weight (g), mean (SD) | 2911(483) | 2960(553) | 2935 (515) |
| SGA, n/N(%)^c^ | 7/25 (28) | 7/24 (29) | 14/49 (29) |
| Apgar-score, median (IQR)^d^ |  |  |  |
| 1-minute | 3(1-3) | 3(2-4) | 3(1-4) |
| 5-minute | 4(4-5) | 6(5-7) | 5(4-7) |
| 10-minute | 7(5-7) | 7(6-8) | 7(6-7,75) |
| pH in cord/blood<60 min, median (IQR), [range]^e^ | 6.81(6.69-6.93) [0.32] | 6.95(6.79-7.08) [0.59] | 6.88 (6.73-7.02) [0.59] |
| BD in cord/blood<60 min, median (IQR), [range]^f^ | 20.0 (22.1-18.7) [10.7] | 16.4 (20.0-12.4) [16.6] | 19.2 (20.9-14.0) [16.6] |
| pH in blood 1-6h, median (IQR), [range]^g^ | 7.25 (7.13-7.36) [0.41] | 7.24 (7.17-7.32) [0.36] | 7.25 (7.15-7.34) [0.41] |
| BD in blood 1-6h, median (IQR), [range]^h^ | 9.3 (16.5-6.8) [18.4] | 12.7 (15.9-6.7) [23.6] | 10.8 (15.9-6.8) [23.6] |
| Clinical seizures before randomization, n/N (%) | 13/25 (52) | 13/25 (52) | 26/50 (52) |
| HIE-stage, n/N(%) |  |  |  |
| HIE grade 2 | 24/25(96) | 24/25(96) | 48/50 (96) |
| HIE grade 3 | 1/25(4) | 1/25(4) | 2/50 (4) |
| Moderately/severely abnormal MRI, n/N (%)^i^ | 2/23 (9) | 10/23 (44) | 12/46 (26) |
| Adverse outcome, n/N (%)^j^ | 5/24 (21) | 12/23 (52) | 17/47 (36) |

Abbreviations. GA, gestational age; SGA, small for gestational age; HIE, hypoxic-ischemic encephalopathy

^a^1 infant was breech delivery. ^b^2 missing (SC). ^c^Defined as birth weight less than the 10^th^ percentile according to the Intergrowth 21^st^ chart. ^d^1-minute: 8 missing, 5-minute: 7 missing, 10-minute: 22 missing. ^e^Data was unavailable for 13 infants in the TH-group and 7 in the SC-group. ^f^Data was unavailable for 14 infants in the TH-group and 8 in the SC-group. ^g^Data was unavailable for 8 infants in the TH-group and 2 in the SC-group. ^h^Data was unavailable for 8 infants in the TH-group and 2 in the SC-group. ^i^Normal/Mildly abnormal MRI was defined as normal/mild basal ganglia and thalami score and/or normal/mild/moderate white matter score. Moderately/Severely abnormal MRI was defined as moderate/severe basal ganglia and thalami score and/or absent posterior limb of the internal capsule and/or severe white matter score. P-value of 0,007 (Chi^2^-test). ^j^Adverse outcome was defined as BSID (Bayley Scales of Infant and Toddler Development) < 85 (motor/cogn comp score), GMFCS (Gross Motor Function Classification System) 3-5, or death. P-value of 0.025 (Chi^2^-test). 3 infants were lost to follow-up.
